# Plasma Metabolomic and Lipidomic Profiling of Metabolic Dysfunction-Associated Fatty Liver Disease in Humans Using an Untargeted Multiplatform Approach

**DOI:** 10.3390/metabo12111081

**Published:** 2022-11-08

**Authors:** Xiangping Lin, Xinyu Liu, Mohamed N. Triba, Nadia Bouchemal, Zhicheng Liu, Douglas I. Walker, Tony Palama, Laurence Le Moyec, Marianne Ziol, Nada Helmy, Corinne Vons, Guowang Xu, Carina Prip-Buus, Philippe Savarin

**Affiliations:** 1 Sorbonne Paris Nord University, Chemistry Structures Properties of Biomaterials and Therapeutic Agents Laboratory (CSPBAT), Nanomédecine Biomarqueurs Détection Team (NBD), The National Center for Scientific Research (CNRS), UMR 7244, 74 Rue Marcel Cachin, CEDEX, 93017 Bobigny, France; 2 CAS Key Laboratory of Separation Science for Analytical Chemistry, Dalian Institute of Chemical Physics, Chinese Academy of Sciences, Dalian 116023, China; 3 School of Pharmacy, Anhui Medical University, Hefei 230032, China; 4 Department of Environmental Medicine and Public Health, Icahn School of Medicine at Mount Sinai, New York, NY 10029, USA; 5 Université d’Evry Val d’Essonne—Université Paris-Saclay, 91000 Evry, France; 6 Muséum National d’Histoire Naturelle, Unité MCAM, UMR 7245, CNRS, 75005 Paris, France; 7 Department of Pathology, University Hospital Jean Verdier, Assistance Publique-Hôpitaux de Paris, 93140 Paris, France; 8 Department of Digestive and Metabolic Surgery, Jean Verdier Hospital, Paris XIII University—University Hospitals of Paris Seine Saint-Denis, 93140 Paris, France; 9 Université Paris Cité, CNRS, INSERM, Institut Cochin, 75014 Paris, France

**Keywords:** metabolomics, lipidomics, NMR, mass spectrometry, multiblock analysis, metabolic dysfunction-associated fatty liver disease

## Abstract

Metabolic dysfunction-associated fatty liver disease (MAFLD) is a complex disorder that is implicated in dysregulations in multiple biological pathways, orchestrated by interactions between genetic predisposition, metabolic syndromes and environmental factors. The limited knowledge of its pathogenesis is one of the bottlenecks in the development of prognostic and therapeutic options for MAFLD. Moreover, the extent to which metabolic pathways are altered due to ongoing hepatic steatosis, inflammation and fibrosis and subsequent liver damage remains unclear. To uncover potential MAFLD pathogenesis in humans, we employed an untargeted nuclear magnetic resonance (NMR) spectroscopy- and high-resolution mass spectrometry (HRMS)-based multiplatform approach combined with a computational multiblock omics framework to characterize the plasma metabolomes and lipidomes of obese patients without (*n* = 19) or with liver biopsy confirmed MAFLD (*n* = 63). Metabolite features associated with MAFLD were identified using a metabolome-wide association study pipeline that tested for the relationships between feature responses and MAFLD. A metabolic pathway enrichment analysis revealed 16 pathways associated with MAFLD and highlighted pathway changes, including amino acid metabolism, bile acid metabolism, carnitine shuttle, fatty acid metabolism, glycerophospholipid metabolism, arachidonic acid metabolism and steroid metabolism. These results suggested that there were alterations in energy metabolism, specifically amino acid and lipid metabolism, and pointed to the pathways being implicated in alerted liver function, mitochondrial dysfunctions and immune system disorders, which have previously been linked to MAFLD in human and animal studies. Together, this study revealed specific metabolic alterations associated with MAFLD and supported the idea that MAFLD is fundamentally a metabolism-related disorder, thereby providing new perspectives for diagnostic and therapeutic strategies.

## 1. Introduction

Non-alcoholic fatty liver disease (NAFLD) is defined as the presence of steatosis (a deposit of lipid droplets) in at least 5% of hepatocytes according to liver biopsy assessment or imaging in patients who have a history of little to no alcohol consumption at all (limited daily alcohol intake is defined as <20 g for women and <30 g for men) with no other cause of hepatic steatosis [1,2,3,4]. NAFLD has now been renamed as metabolic dysfunction-associated fatty liver disease (MAFLD) [5]. MAFLD is characterized by the abnormal accumulation of lipids, mainly triacylglycerols (TGs), in the liver. Based on clinical–histologic characteristics, the MAFLD spectrum ranges from simple fatty liver or steatosis (ST) to an advanced form termed non-alcoholic steatohepatitis (NASH). Without therapeutic intervention, some patients with NASH subsequently progress to cirrhosis and, ultimately, hepatocellular carcinoma [6]. In parallel with the emerging epidemic of metabolic syndromes (insulin resistance, obesity, diabetes, etc.), MAFLD has emerged as a leading global cause of chronic liver disease in the past few decades, with a prevalence of more than 25% in the global adult population; however, this ratio has been estimated to be over 30% in France [7,8].

Despite its growing prevalence, the factors involved in the development of MAFLD and its subsequent progression to NASH, fibrosis, cirrhosis and hepatocellular carcinoma are largely unknown. It is well established that the etiology of MAFLD is multifactorial, with important risk factors that include a number of gene polymorphisms (such as those in patatin-like phospholipase domain-containing protein 3 (*PNPLA3*), transmembrane 6 superfamily member 2 (*TM6SF2*) and glucokinase regulatory protein (*GCKR*), which have been shown to be associated with MAFLD and its severity [9,10,11]), as well as dietary factors (e.g., fructose), insulin resistance [12], obesity, type 2 diabetes mellitus, hyperlipidemia, environmental exposure to endocrine disruptors (including perfluorinated alkyl substances (PFAS) [13,14]) and gut microbiota dysbiosis [15,16].

Currently, liver biopsy is the gold standard for MAFLD diagnosis, staging and monitoring progression during treatments. Nevertheless, biopsies have well-known limitations, including sampling variability, invasiveness, poor acceptability among patients and relatively high financial costs for post-biopsy patient care, all of which reduce their applicability to large populations. Additionally, recently developed non-invasive imaging biomarker assessment strategies, including even the most accurate non-invasive liver elastography-based methods (such as vibration-controlled transient elastography (VCTE), magnetic resonance elastography (MRE), shear wave elastography and acoustic radiation force impulse), have other limitations, such as the inability to assess inflammation and limited available user guidance regarding how to anticipate and manage the pitfalls of these tests [17]. Thus, there is an urgent need to develop non-invasive and clinician-friendly strategies, such as using non-invasive biomarkers [7] for the prognostication, staging and selection of patients for the treatment and monitoring of MAFLD.

Untargeted metabolomics and lipidomics have the potential to analyze a larger spectrum of small chemical metabolites or lipids in biomedical samples, which can provide a comprehensive view of the metabolic perturbations of MAFLD [18]. Previous studies have suggested that plasma metabolomes could be better predictors for steatosis (with a predictive power of 80%) than clinical data (with a predictive power of 58%) [19,20]. Nuclear magnetic resonance (NMR) spectroscopy and liquid chromatography–mass spectrometry (LC–MS) are two robust analytical platforms used for metabolomics and lipidomics [21]. Thus, integrating their complementary advantages in detection coverage, sensitivity and the resolution and identification of unknown compounds could enhance the detection coverage and identification abilities of untargeted metabolomics and lipidomics. To the best of our knowledge, the use of metabolomic and/or lipidomic profiling with both NMR and LC–MS platforms on biopsy confirmed MAFLD patients is still very limited [18,22]. In this study, we performed plasma metabolomic and lipidomic profiling using an untargeted NMR- and mass spectrometry-based multiplatform approach on a cohort of morbidly obese patients with different stages of MAFLD. The objectives of our study were three-fold: (1) describe the relative plasma metabolome and lipidome changes in ST and NASH compared to health control (HC) obese patients; (2) investigate whether plasma metabolomic and lipidomic analysis could help to uncover potential metabolic markers or metabolic pathways associated with MAFLD, if any; (3) identify clinical, metabolomic and lipidomic patterns associated with the disease with the help of a computational multiblock omics framework.

## 2. Materials and Methods

### 2.1. Patients and Biological Samples

During June 2011 and May 2015, a total of 97 obese patients undergoing bariatric surgery were recruited from the digestive and metabolic surgery service at the Jean Verdier University Hospital (Bondy, France). All adult obese patients fulfilled the eligibility criteria for bariatric surgery according to the French High Authority of Health recommendations: (1) a body mass index (BMI) ≥ 40 or ≥35 with at the least one associated comorbidity (dyslipidemia, hypertension arterial disease, obstructive sleep apnea syndrome, osteoarthritis, type 2 diabetes and/or NAFLD); (2) physical and psychological eligibility for a bariatric surgical procedure (decided in multidisciplinary consultation meetings at the surgery service). Autoimmune, inflammatory or infectious liver diseases (including viral hepatitis, hemochromatosis, previous chemotherapy, hepatotoxic treatment and known alcohol consumption (over 20 g/day for women and over 30 g/day for men)) were exclusion criteria. All patients involved in the study signed informed consent documents for participation in biomedical research. The study was conducted in accordance with the French “Huriet-Sérusclat law” and the Helsinki Declaration, and approved by the local ethics committee of the Protection of Persons (CPP) of Ile de France X (ethic approval code: 2010-02-02, approval date: 11 February 2010). Pre-operative blood plasma samples were collected from patients. Deep parenchymal liver biopsies were taken from the left hepatic lobes of the obese patients via laparoscopy, which was performed at the beginning of the operation before any liver manipulation to avoid tissue trauma and electrocoagulation. A histological analysis of formalin-fixed and paraffin-embedded liver sections stained with hematoxylin–eosin and picrosirius red was performed by a pathologist who specializes in liver pathology. The FLIP algorithm described by Bedossa et al. [23] was used to classify the liver biopsies into three categories: health control (HC; steatosis, inflammation and ballooning = 0); steatosis (ST; steatosis 1–3 with either inflammation 1–3 or ballooning 1–2); NASH (steatosis 1–3 with 1–3 inflammation and 1–2 ballooning ± 1–4 fibrosis). The liver TG contents were measured using a colorimetric diagnostic kit (Triglycerides FS; DiaSys Diagnostic Systems GmbH) following the extraction of the total liver lipids using chloroform/methanol (2:1, *v*/*v*) and their separation via thin-layer chromatography on silica gels plates (Merck KGaA, Darmstadt, Germany) [24].

### 2.2. Sample Preparation and NMR- and MS-Based Analysis

NMR profiling was accomplished with a 500 MHz Bruker AVANCE III ^1^H NMR spectrometer (Advance III, Bruker, Germany) on an automatic sample changer with cooling capability. The NMR spectra were acquired via two complementary experiments on each plasma sample: one-dimensional (1D) ^1^H nuclear Overhauser effect spectroscopy NOESY1D presat (NOESY1dgppr sequence) [25] and Carr–Purcell–Meiboom–Gill (CPMG presat) [26]. The spectral width was set at 10 kHz. A Fourier transformation was carried out on the NMR data with the line broadening (LB) set at 0.3 Hz. The spectra were phased and baseline corrected, then all spectra were aligned on a glucose doublet at 5.23 ppm. Finally, each 1D NMR spectrum was sliced into buckets of 0.001 ppm, containing NMR signals. These steps were accomplished using NMRPipe software [27]. Before the statistical analysis, the NMR spectra bins were normalized to the median of intensities [28]. The assignment of the ^1^H NMR spectra was performed using two-dimensional (2D) experiment, Chenomx software and the Human Metabolome Database (HMDB) (version 4.0) [29].

Raw MS data were collected, converted to mzML format files using ProteoWizard software [30] and processed in each ionization mode (ESI+ or ESI−) as a single batch using the XCMS pipeline [31], which was implemented on MetaboAnalyst 5.0 [32]. This generated MS feature tables. The features were filtered to remove those with more than 50% non-detected responses in samples before further analysis. The missing values were replaced with the average of the corresponding non-missing values in the k (here, k = 5) closest features in terms of the Euclidean distance of the responses across all samples. After processing, the MS feature responses were log_2_ transformed and used for further analysis. Additional details are provided in the Supplementary Materials.

### 2.3. Data Analysis

The processed 1D ^1^H NOESY NMR data were used for multivariate analysis. An orthogonal partial least squares discriminant analysis (OPLS-DA) was carried out using an in-house MATLAB OPLS script, which was based on the Trygg and Wold method [33]. The statistical analysis and visualization were performed on MATLAB^®^ R2016b for macOS (Mathworks, Natick, MA, USA). The quality parameters of the models, the explained variance (R2Y) and the predictability of the model (Q2Y) were calculated. Q2Y was computed via 7-fold cross-validation and confirmed by exploring the impacts of permutations in the dataset rows.

Variables missing more than 30% of values in the clinical data were removed from analysis and the missing values were imputed using the predictive mean matching method with the R package *mice* [34]. Box and whisker plots were generated using the R packages ggplot2 (https://ggplot2.tidyverse.org/, accessed on 1 October 2022) and ggprism [35].

A metabolome-wide association study (MWAS) was accomplished using a linear regression framework with the R package xmsPANDA (https://github.com/kuppal2/xmsPANDA, accessed on 1 October 2022). For each feature, the log_2_ transformed response was used to test the relationships across the different stages of MAFLD, including HC, ST and NASH, as continuous variables (HC = 0, ST = 1 and NASH = 2). The model included adjustments for age (continuous) and gender (categorical), which are known to influence plasma metabolomes [36,37]. To account for the multiple comparisons, Benjamini and Hochberg-based false discovery rate (FDR) thresholds of 0.2 and 0.25 were applied for lipidomics data and metabolomic data, respectively. A enriched metabolic pathway analysis was carried out using Mummichog [38], which was implemented on MetaboAnalyst 5.0 [32]. Briefly, the process was based on low confidence matches to determine compounds by predicting chemical formulae using untargeted LC–MS-based metabolomic data. This improved our ability to generate hypotheses regarding metabolic pathway alterations associated with MAFLD. Positive and negative mode data were concatenated as a single matrix using the mixed mode method. All features, including retention time, were used for the Mummichog analysis to estimate the null model (background) and improve feature annotation. Annotations were assigned using the HumanMFN metabolic model [32] and the evidence scoring provided in Mummichog. Identities were assigned using the evidence scoring for matching to adducts at a ±5 ppm mass tolerance for metabolomic data and a ±10 ppm for lipidomics data, with the scoring threshold set to 0.05. Metabolic activity patterns associated with MAFLD were characterized using Mummichog. A multiblock analysis was carried out using the R package mixOmics [39]. The metabolomic and lipidomic features and clinical characteristics selected by MWAS were included in the multiblock model to identify highly correlated multi-omics signatures that discriminated the MAFLD stages: HC, ST and NASH. Additional details are provided in the Supplementary Materials. 

## 3. Results

### 3.1. Study Design and the Characteristics of HC, ST and NASH Patients

**Figure 1:**
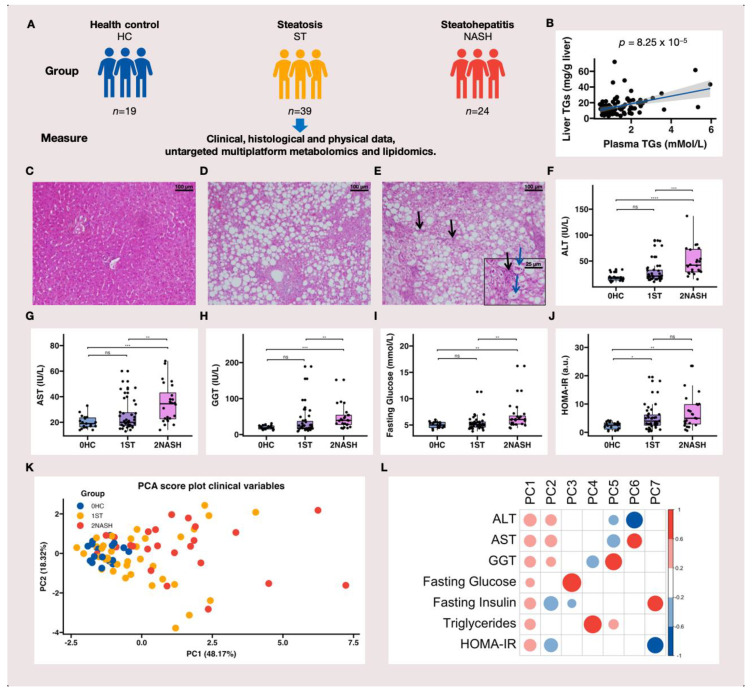
The associations between clinical characteristics and MAFLD: (**A**) a simplified diagram of the study design; (**B**) the correlation between plasma and liver TGs; (**C**–**E**) the results of the liver histological analysis using hematoxylin–eosin staining on the different groups of patients ((**C**) a normal liver (HC) from a 30-year-old woman, magnification 100× (scale bars: 100 μm); (**D**) steatosis (ST) from a 30-year-old woman showing “isolated” macrovacuolar steatosis affecting 60–90% of hepatocytes without inflammation, ballooning and fibrosis, magnification 100× (scale bars: 100 μm); (**E**) non-alcoholic steatohepatitis (NASH) from a 40-year-old woman showing macrovacuolar steatosis affecting 60–90% of hepatocytes with inflammation (black arrows) and ballooning (blue arrows), magnification 100× (scale bars: 100 μm), inset 400× (scale bars: 25 μm)); (**F**–**J**) the clinical and biochemical characteristics of the patients ((**K**) the PCA results for all participants based on seven clinical characteristics; (**L**) a correlation matrix highlighting the most significant contributing variables for each principal component. The Benjamini and Hochberg adjusted *p*-values were computed using Dunn’s multiple comparison test [42], which is a post-hoc Kruskal–Wallis test. Note: ns, non-significant; * *p* < 0.05; ** *p* < 0.01; *** *p* < 0.001; **** *p* < 0.0001; AST, aspartate transaminase; ALT, alanine transaminase; GGT, gamma-glutamyl transferase; HOMA-IR, homeostatic model assessment for insulin resistance; TGs, triglycerides; HC, health control (normal liver); ST, non-alcoholic fatty liver or steatosis; NASH, non-alcoholic steatohepatitis; a.u., arbitrary unit; PC, principal component.

A simplified diagram of the study design is shown in Figure 1A. The NMR data and the LC–MS-based metabolomic and lipidomic data were collected from the baseline plasma of 85 (87.6%), 80 (82.5%) and 82 (84.5%) patients, respectively, due to the limited number of plasma samples. Among the 82 patients (lipidomics), there were 66 females (80%) and 16 males (20%) (Table S1). The diagnoses of HC (Figure 1C), ST (Figure 1D) and NASH (Figure 1E) were established histologically using liver biopsy specimens (Figure 1C–E). Figure 1B shows the positive correlation between plasma and liver TGs, demonstrating that elevated triglyceride levels, in addition to liver TG content, could be a key feature of MAFLD in obese patients. The clinical and biochemical characteristics of the patients are shown in Figure 1F–J and Table S1 in the Supplementary Materials. Patients with NASH had significantly higher levels of alanine aminotransferase (ALT) (Figure 1F), aspartate aminotransferase (AST) (Figure 1G), gamma-glutamyl transferase (GGT) (Figure 1H), plasma TGs (Table S1) and fasting blood glucose (Figure 1I) compared to both HC and ST patients. Compared to obese HC patients, patients with MAFLD (ST and NASH) were more likely to have insulin resistance (Figure 1I,J) [40] and significantly higher levels of plasma TGs (Table S1). There were no significant differences in body mass index (BMI) or plasma cholesterol between patients with ST and those with NASH (Table S1). In concordance with a previous study on MAFLD [41], there was a tendency for these seven characteristics (ALT, AST, GGT, fasting insulinemia, fasting glycemia, HOMA-IR and plasma TG content) to increase in patients with ST or NASH, suggesting their importance in MAFLD progression. We then performed a principal component analysis (PCA), as shown in Figure 1K. There was a high degree of separation between patients with HC, ST and NASH. ALT, AST, GGT, fasting insulin and plasma triglyceride levels strongly contributed to the classification (Figure 1L), which suggested a high correlation with MAFLD. Again, our results were consistent with the findings of another large MAFLD cohort study (*n* = 679) [23]. Therefore, these five characteristics were used for our multiblock analysis.

### 3.2. Metabolomic and Lipidomic Profiles of HC, ST and NASH Patients

**Figure 2:**
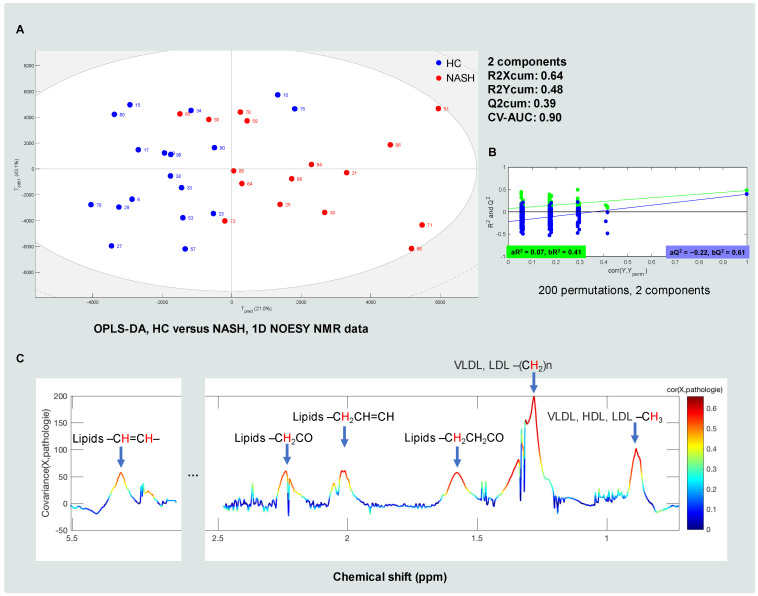
The NMR-based plasma metabolomics of MAFLD: (**A**) the OPLS-DA score plot (HC versus NASH; 1D NOESY data); (**B**) the 200 times permutation test for the OPLS-DA model (HC versus NASH; 1D NOESY data); (**C**) the OPLS-DA covariance plot (HC versus NASH; 1D NOESY data). The red hydrogen atom indicates the ^1^H detected by ^1^H NMR.

The OPLS-DA models for the classification of HC, ST and NASH patients were initially investigated using the NMR data. Because the HC and NASH patients represented the normal and advanced stages of MAFLD, respectively, they displayed the maximum difference regarding the stage of the disease; hence, we initially tested the classification models on these two groups. An OPLS-DA model with two components (one predictive component and one orthogonal component) was obtained. The score plot of the model is represented in Figure 2A. To estimate performance, the models were evaluated by a 200 times permutation test (as represented in Figure 2B) and repeated (*n* = 200) 7-fold cross-validated area under the ROC curve (CV-AUC) tests, with an R2Xcum of 0.64, an R2Ycum of 0.48 (R2 is the indicator of how the model fits the data; the closer to 1, the better the fit) and a Q2cum of 0.39 (Q2 is the capacity of the model to correctly class a new dataset; the closer to 1, the better the model predictability). The CV-AUC was 0.896, which meant the probability that the OPLS-DA model scored a randomly chosen NASH patient who was classed as a NASH patient was 89.6% higher than a randomly chosen NASH patient who was classed as an HC patient. All of which indicated that our OPLS-DA model for the classification of HC and NASH patients was fairly stable. The model variables that contributed most significantly to the classification of HC and NASH patients are represented in Figure 2C. An OPLS-DA covariance plot was generated, which restored the form of the NMR spectra and colored them according to their correlation scores with NASH). The red spectra regions had a >0.5 correlation with NASH.

As represented in Figure 2C, the major discriminant compounds that could differentiate HC patients from NASH patients were lipids, including VLDL, LDL and HDL. OPLS-DA models that classified HC and ST patients as well as ST and NASH patients were also investigated, but we did not find any significant models for the classification of HC and ST patients nor for the classification of ST and NASH patients after several tests. In brief, the first part of the NMR-based metabolomic analysis demonstrated that lipids, such as VLDL, LDL and HDL, were important signatures that could discriminate between HC and NASH patients. The next step was then to identify which lipids or lipid classes contributed the most to the classification of patients. As we know that these metabolites are important in the disease, it was logical to suggest that some lipids may help in discriminating between HC, ST and NASH patients. Thus, we performed UHPLC-HRMS-based metabolomic and lipidomic analyses to cover a larger number of lipids and metabolites.

### 3.3. Metabolome-Wide Association Study (MWAS) of MAFLD

**Figure 3:**
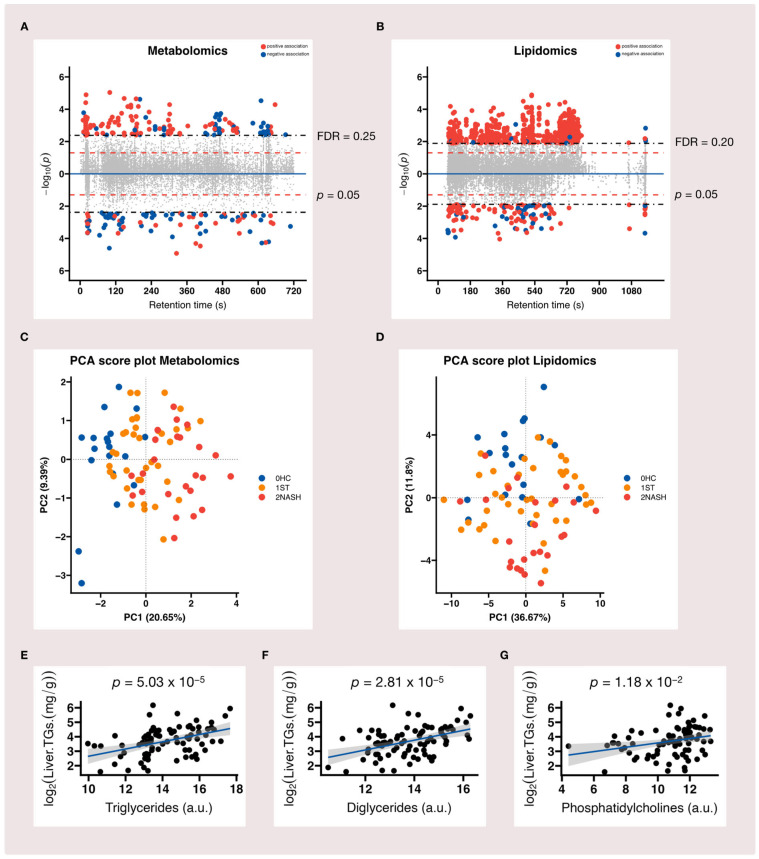
Our MAFLD metabolome-wide association study: (**A**) MWAS highlighted 241 metabolomic features associated with the disease at FDR < 25%. (**B**) 787 lipidomic features were associated with the disease at FDR < 20%. The figure represents the retention time (s) of LC-MS features detected in positive (above the blue line) and negative (below the blue line) ionization mode plotted against −log_10_(*p*-value), respectively. Each dot represents a unique LC-MS feature: red dots are features that positively associated with MAFLD, while blue dots are features that negatively associated with MAFLD. The “dashed” red horizontal line indicates the raw *p*-Value of 0.05 and the “dot–dash” horizontal line shows the false discovery rate-adjusted *q*-Value of 0.2 for lipidomics and 0.25 for metabolomics; (**C**,**D**) the PCA score plots for the metabolomic and lipidomic data, respectively; (**E**–**G**) the three main lipids that matched the top 10 ranked features based on the regression *p*-value and correlation with liver TG content.

We focused on endogenous metabolic pathway perturbations that are associated with MAFLD to generate insights into the systemic biological changes underlying the pathogenesis of the disease. In total, 14,678 LC–MS features (10,750 in positive mode and 3928 in negative mode) were detected in our metabolomic analysis, while 12,278 LC–MS features (8791 in positive mode and 3487 in negative mode) were detected in our lipidomic analysis. After we applied the primary feature selection criteria with the linear regression model raw *p*-value of <0.05 and the Benjamini and Hochberg (BH) method-based false discovery rate-adjusted *q*-value of <25%, 241 LC–MS features (148 in positive mode and 93 in negative mode) were found to be significant in the metabolomic analysis (Figure 3A), while 787 features (659 in positive mode and 128 in negative mode) were found to be significant (with a raw *p*-Value of <0.05 and a false discovery rate-adjusted *q*-Value of <20%) in the lipidomic analysis (Figure 3B).

We initially computed a PCA of selected features and there was tendency that the NASH group was different from the HC group in terms of both metabolomic and lipidomic data; however, the ST group was close to both the HC and NASH groups (Figure 3C,D). The first principal component represented the largest variation between groups and the percentage of variances explained by the first principal component was higher for the lipidomic data (36.67%) than the metabolomic data (20.65%). We then generated heatmaps of the first 25 features ranked by *p*-values. As represented in Figure S2A,B in the Supplementary Materials, these features were positively correlated with the HC group and negatively correlated with the NASH group. While the correlations with the ST group were more mixed, there were different patterns: some of the features were slightly positively correlated with the ST group, while others were slightly negatively correlated with the ST group. This suggested the presence of subgroups within the ST group, which was in concordance with another previous study on MAFLD [43]. The annotations of the top 10 ranked features based on the regression *p*-Value matched to three main lipids (Figure 3E–G) and they were well correlated with liver TG content.

### 3.4. Classification of HC, ST and NASH Patients Based on MS Data

**Figure 4:**
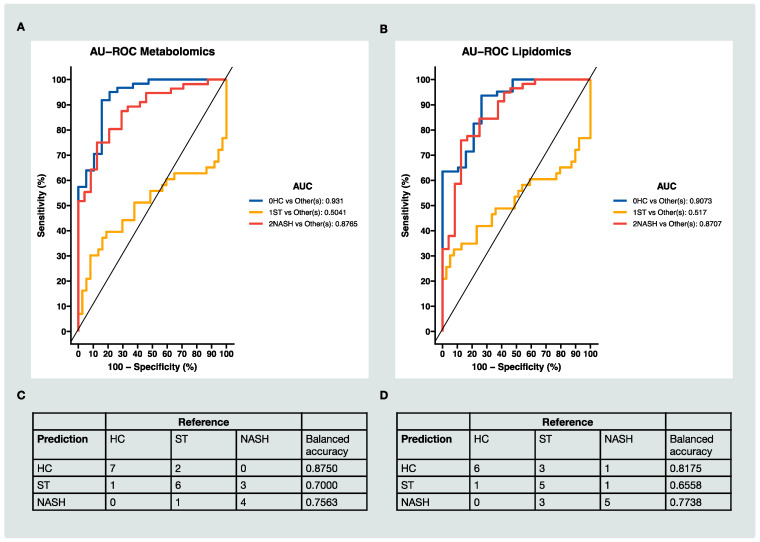
The classification of HC, ST and NASH patients based on MS data: (**A**) the ROC curve and AUC from the sPLS-DA for the metabolomic data of the first component; (**B**) the ROC curve and AUC from the sPLS-DA for the lipidomic data of the first component; (**C**) the confusion matrix of the test set sample (*n* = 24) from the sPLS-DA for the metabolomic data; (**D**) the confusion matrix of the test set sample (*n* = 25) from the sPLS-DA for the lipidomic data. Note: balanced accuracy = (sensitivity + specificity)/2.

We then investigated the supervised sparse partial least squares discriminant analysis (sPLS-DA) method, which was based on the partial least squares regression (PLS) for discrimination analysis, and a Lasso penalization was applied to select the optimal variables [44]. The sPLS-DA results showed that the optimal value for components was 1 in both the metabolomic data (Figure S1A) and lipidomic data (Figure S1B). The optimal values for variables were 240 (Figure S1C) and 3 (Figure S1D) in metabolomic and lipidomic data, respectively. Among the selected metabolomic features, nearly half were positively correlated with the stage of MAFLD and nearly half of them were negatively correlated with the stage of MAFLD (Figure S2A). The selected lipidomic features were negatively correlated with the stage of MAFLD (Figure S2B). The area under the receiver operating characteristic curve (AU-ROC) and a confusion matrix were computed to evaluate the prediction accuracy of the model. The outcomes on metabolomic data (Figure 4A) and lipidomic data (Figure 4B) were comparable. Both sets of data showed a good classification for HC patients versus the other groups (AUC > 0.90) and for NASH patients versus the other groups (AUC > 0.87). However, it was difficult to distinguish the ST group from the others. The confusion matrix of the test set sample (a random subset comprising 30% of the total samples: *n* = 24 for metabolomic data and *n* = 25 for lipidomic data) from the sPLS-DA also showed comparable results for the metabolomic data (Figure 4C) and lipidomic data (Figure 4D).

### 3.5. Multiblock Integrative Analysis of MAFLD

**Figure 5:**
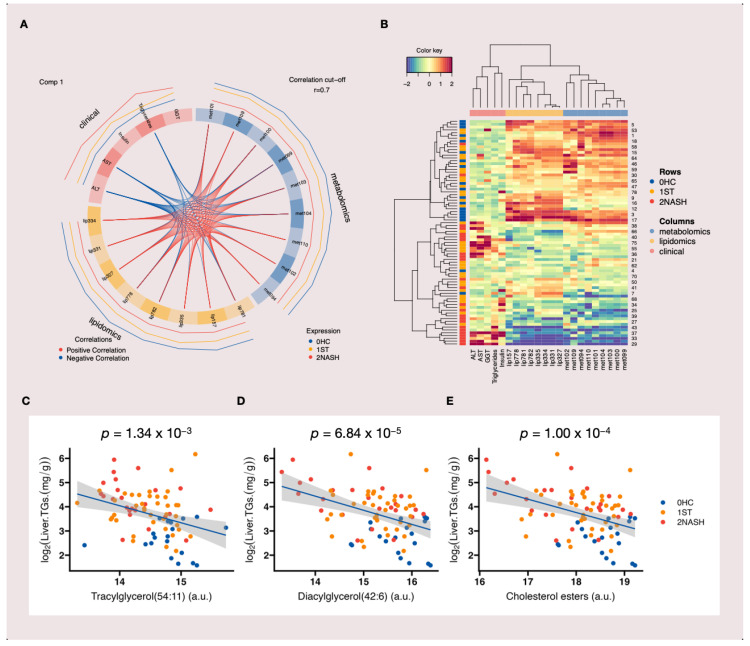
Our multiblock integrative analysis for clinical, metabolomic and lipidomic data: (**A**) the circos plot showing the integrative frameworks and the positive (red lines) and negative (blue lines) correlations (cutoff r = 0.7) between selected variables from each block, represented on the side quadrants (the selected feature names were coded as “metxxx” for metabolomic data and “lipxxx” for lipidomic data); (**B**) the clustered image map (CIM) for the variables selected by our multiblock sPLS-DA of the first component in which the Euclidean distance and complete linkage methods were used (the CIM shows the samples in the rows (as indicated by their stages of MAFLD on the left-hand side of the plot) and the selected features in the columns (as indicated by their data type at the top of the plot); (**C**–**E**) the three main lipids that matched the selected features from the multiblock analysis and correlation with liver TG content. Note: a.u., arbitrary unit.

To identify highly correlated multi-omics markers that could discriminate between HC, ST and NASH patients, we performed a multiblock analysis [39] by integrating clinical, metabolomic and lipidomic data (Figure S3A). The circos plot in Figure 5A shows the correlations between the variables of different blocks, represented on the side quadrants. It can be seen that the clinical variables were almost entirely negatively correlated with the other two DataFrames (metabolomic and lipidomic data), while the metabolomic and lipidomic variables were more mixed and were positively correlated with each other. The correlation cutoff was set at 0.7 (cutoff = 0.7). As shown in Figure S3A, the first components from the metabolomic and lipidomic datasets and the lipidomic and clinical datasets were highly correlated with each other (as indicated by the large numbers in the bottom left of the figure). In the figure, the colors and ellipses related to the stages of MAFLD indicate the discriminative ability of each component to separate the different stages of MAFLD. The selected metabolomic and lipidomic features showed similar profiles among different stages of MAFLD and they had high levels of expression in the HC group and low levels of expression in the NASH group (Figure 5B and Figure S3). These features could discriminate between the HC and NASH groups. The clinical variables were more likely to have high levels of expression in the NASH group (Figure 5B and Figure S3). For the selected metabolomic and lipidomic features, it seemed that the levels of expression in the ST group showed different patterns: some of them showed similar profiles to those in the HC group, while others showed similar profiles to those in the NASH group (Figure 5B). These results supported the previous hypothesis of the presence of subgroups in the ST group. The annotation of the selected features in the multiblock analysis matched to three main lipids (Figure 5C–E) and they were well correlated with liver TG content.

### 3.6. Enriched Metabolic Pathways Associated with MAFLD

To uncover the potential biological mechanisms underlying the MAFLD-associated MS features from our MWAS, pathway enrichment analyses were performed using Mummichog, which directly predicts metabolic activity from HRMS data without a priori MS feature identification. Mummichog has been shown to improve biological interpretation while balancing false positive and false negative errors in the enrichment of high-dimensional mass spectral data [45]. Our Mummichog analysis identified the significant enrichment of 16 pathways (Table 1 and Table 2) associated with MAFLD: 7 were identified in both the metabolomic and lipidomic analyses, while 9 of them were only detected in one type of analysis (1 in the metabolomic analysis and 8 in the lipidomic analysis). The highlighted pathway changes included steroid metabolism, fatty acid metabolism, carnitine shuttle, amino acid metabolism, glycerophospholipid metabolism and arachidonic acid metabolism. These related to biological processes in liver function (bile acid metabolism), energy metabolism (carbohydrate metabolism, lipid metabolism and amino acid metabolism), cell proliferation (nucleotide metabolism and phospholipid metabolism), antioxidant defense (the metabolism of vitamin E) and inflammatory response (arachidonic acid metabolism and steroid metabolism).

## 4. Discussion

In this study, we performed plasma metabolomic and lipidomic profiling among biopsy confirmed MAFLD patients using an untargeted NMR- and HRMS-based multiplatform approach to uncover changes in metabolic pathways by identifying disease-related patterns and biochemical perturbations to generate a comprehensive view of potential MAFLD pathogenesis in humans. Our results revealed significant changes in several key pathways linked to MAFLD, specifically amino acid, arachidonic acid, bile acid and lipid metabolism.

**Figure 6:**
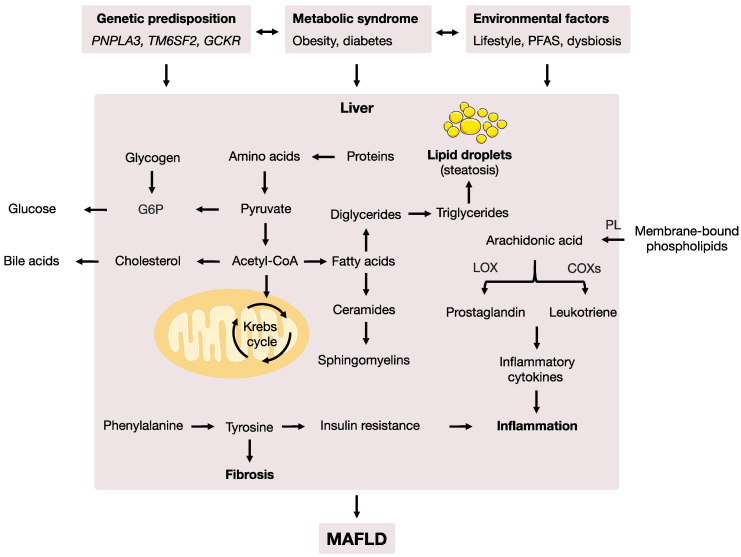
The potential cross-talk between different factors and biological pathways involved in the development of MAFLD. MAFLD is a complex disease that involves dysregulations in multiple biological pathways, specifically amino acid, arachidonic acid, bile acid and lipid metabolism, which is modulated by interactions between genetic predisposition, metabolic syndromes and environmental factors. Note: acetyl-CoA, acetyl coenzyme A; G6P, glucose 6-phosphate; PFAS, perfluorinated alkyl substances; COXs, cyclooxygenases; LOXs, lipoxygenases; PL, phospholipase; *PNPLA3*, patatin-like phospholipase domain-containing protein 3 gene; *TM6SF2*, transmembrane 6 superfamily member 2 gene; *GCKR*, glucokinase regulatory protein gene. Partially inspired by Masoodi et al. [18]. The mitochondrion and lipid droplets illustrations were adapted (modified with layer and text) from Servier Medical Art. Servier Medical Art by Servier is licensed under a Creative Commons Attribution 3.0 Unported License (https://creativecommons.org/licenses/by/3.0/, accessed on 1 October 2022).

Amino acids are one of the substrates for gluconeogenesis [46] and liver and skeletal muscles are the primary sites of amino acid metabolism. Significant changes in amino acid metabolism suggest that changes in protein turnover are likely a consequence of increased insulin resistance and protein catabolism [47]. Tyrosine is a non-essential aromatic amino acid that is primarily metabolized in the liver and our body can produce it from phenylalanine (Figure 6). Previous studies have suggested that tyrosine is associated with insulin resistance and fibrosis. Similar findings have been reported in other types of chronic liver disease [46,48]. In a study by Kalhan et al., the results showed that phenylalanine was elevated in subjects with NASH but not in subjects with steatosis. The absence of an increase in essential amino acids, including phenylalanine, in the steatosis stage suggests that changes in protein turnover may be a late event in the progression from steatosis to NASH and may be modulated by other factors, such as cytokines and inflammation, in addition to insulin resistance [49]. 

Arachidonic acid (Figure 6) is an essential fatty acid. It is released from membrane-bound phospholipids by phospholipase (PL) enzymes and metabolized by cyclooxygenases (COXs) and lipoxygenases (LOXs) into the pro-inflammatory compounds (known as cytokines) prostaglandin and leukotriene, respectively [50,51]. Significant changes in arachidonic acid metabolism suggest altered inflammatory responses, combined with insulin resistance, which can modulate the progression of MAFLD.

Bile acids are steroids that are synthesized by the liver from cholesterol. These amphiphilic molecules play important roles in metabolism (including facilitating the absorption of fat from diet and lipophilic vitamins in the small intestine) as signaling molecules that regulate energy expenditure, glucose, lipid metabolism and inflammation via the nuclear farnesoid X receptor (FXR) and the Takeda G protein-coupled receptor 5 (TGR5) [52,53]. Bile acid metabolism is an indicator of liver function. In a study by Grzych et al., the results showed that changes in plasma bile acid levels depended on insulin resistance. Combined with our results, significant changes in bile acid metabolism suggest altered liver function, metabolism, inflammation and insulin resistance.

MAFLD is characterized by the abnormal accumulation of lipids (mainly TGs) in the liver. The genetic variants associated with MAFLD risk are heavily linked to genes involved in the modulation of lipid metabolism [9,10,54]. Thus, lipid metabolism plays an important role in the progression of the disease. In our study, significant changes in de novo fatty acid biosynthesis, fatty acid activation and fatty acid metabolism suggested dysregulation in the pathways involved in the modulation of TG metabolism (Figure 6). Together, the results from the enriched metabolic pathway analysis in our study were in good agreement with findings from previous studies and suggested dysregulations in fundamental metabolism pathways during the development of MAFLD.

MAFLD is becoming the most common chronic liver condition in the world; however, the diagnosis of MAFLD remains challenge and there is an urgent need for non-invasive biomarkers for prognostication and the selection of patients for the treatment and monitoring of the disease [7]. In our study, our MWAS (Figure 3) and multiblock integrative analysis (Figure 5) revealed several small molecular features (Figure 3E–G and Figure 5C–E) that were strongly associated with the disease, which could be promising metabolic markers for MAFLD. The differences between the ST and NASH stages are inflammation, fibrosis and liver cell death [55]. It was logical to suggest that these selected features were negatively correlated with these biological processes (Figure 5B and Figure S3B) and thus, could have protective effects against MAFLD. In the classification of HC, ST and NASH patients, lipidomic and metabolomic analysis results from our study were concordant. Nevertheless, there were more significant features that were associated with MAFLD in the lipidomic analysis (787) than the metabolomic (241) analysis (Figure 3A,B). The outcomes of the classification model, including the AU-ROC and confusion matrix, were slightly better from the lipidomic analysis than the metabolomic analysis (Figure 4). The distinction between ST patients and HC patients was difficult in our study due to the subjects being in the advanced stages of obesity with very high BMI scores and insulin resistance. Similar results have been reported previously by Kalhan et al. [49].

The strengths of the present study were the combination of the robust untargeted NMR- and UHPLC-HRMS multiplatform-based metabolomic and lipidomic analyses of biopsy confirmed samples using a computational multiblock omics framework. Nevertheless, our study had limitations. Firstly, the subjects were obese with high BMI scores, which could have impacted metabolism, such as lipid metabolism, thereby complicating the interpretation of our results. There have been studies that have compared metabolism changes in healthy subjects and non-obese MAFLD patients. He et al. showed that some acylcarnitine could discriminate NAFLD patients from healthy control patients [56]. Wang et al. noticed that many lipids (PC, SM and PE) were elevated in NASH patients compared to healthy control patients [57]. It could be interesting to design new cohort studies to investigate how the metabolic differences underlined here compare to those from previous findings. Secondly, it was a modest-size cohort; therefore, the number of patients in each group may not have been enough to achieve strong significant statistic outcomes. Still, our results were in good agreement with previous studies on MAFLD among large cohorts [23,41].

## 5. Conclusions

In conclusion, the results of this study suggested that plasma metabolomic and lipidomic analyses, especially lipidomic analysis, could be promising approaches for identifying disease-related patterns and biochemical perturbations caused by MAFLD. Our results revealed significant changes in several key metabolic pathways linked to MAFLD, specifically amino acid, arachidonic acid, bile acid and lipid metabolism, even though the roles of some clinical features could not be fully excluded. Interestingly, in our results, it could be seen that the metabolomic and lipidomic approaches confirmed some biochemical results, pointing out the roles of particular DGs or TGs in the metabolic pathways linked to MAFLD. Our multiblock integrative analysis demonstrated that the concentration of several sphingomyelins and diacylglycerols was correlated to the concentrations of several phosphatidic acids and acetylspermidine and were not correlated with the concentration of TGs in the blood. Further studies are necessary to fully determine the dysregulation of the metabolic pathways, which could provide a comprehensive view of potential MAFLD pathogenesis in humans. Together, our results supported that MAFLD is fundamentally a metabolism-associated disorder. Nevertheless, it should be mentioned that MAFLD is a heterogeneous and complex multi-organ disease [49], so further investigation should particularly focus on lipidomics, as well as the subtypes of patients. Moreover, integrating other omics approaches (including transcriptomics, proteomics, exposomics and clinical biochemical characteristics) could improve the novel subtyping of MAFLD patients (thereby allowing the precise classification of patients), improve our understanding of the biochemical processes behind the disease, generate insights into the systemic biological changes underlying the pathogenesis of MAFLD in humans and develop personalized precision medicine-based strategies for the prevention, therapy and management of MAFLD.

## Figures and Tables

**Table 1:** The enriched metabolic pathways associated with MAFLD (metabolomics).

Metabolic Pathway	Significant Overlap ^1^	Adj. *p*-Value ^2^	Biological Process
Tyrosine metabolism	5	1.87 × 10^−3^	Amino acid metabolism
Linoleate metabolism	5	4.86 × 10^−4^	Lipid metabolism
Fatty acid activation	4	5.26 × 10^−4^	
Fatty acid Metabolism	3	6.15 × 10^−4^	
De novo fatty acid biosynthesis	4	6.20 × 10^−4^	
Purine metabolism	3	8.29 × 10^−4^	Nucleotide metabolism
Glycosphingolipid metabolism	3	6.31 × 10^−4^	Phospholipid metabolism
Glycerophospholipid metabolism	3	4.54 × 10^−3^	

^1^ Number of significant metabolites; ^2^ the adjusted *p*-Values were calculated to estimate the false discovery rate. Pathways with ≥3 significant metabolites and FDRs of <25% were considered.

**Table 2:** The enriched metabolic pathways associated with MAFLD (lipidomics).

Metabolic Pathway	Significant Overlap ^1^	Adj. *p*-Value ^2^	Biological Process
Tyrosine metabolism	3	2.61 × 10^−3^	Amino acid metabolism
Arachidonic acid metabolism	6	8.81 × 10^−4^	Arachidonic acid metabolism
Prostaglandin formation from arachidonate	5	1.24 × 10^−3^	
Leukotriene metabolism	5	1.79 × 10^−3^	
Glycerophospholipid metabolism	12	7.07 × 10^−4^	
Glycosphingolipid metabolism	3	2.10 × 10^−2^	
Bile acid biosynthesis	4	5.15 × 10^−2^	Bile acid metabolism
C21-steroid hormone biosynthesis and metabolism	7	3.09 × 10^−3^	
Sialic acid metabolism	3	3.20 × 10^−3^	Carbohydrate metabolism
Fatty acid activation	6	6.85 × 10^−4^	Lipid metabolism
De novo fatty acid biosynthesis	7	8.03 × 10^−4^	
Fatty acid Metabolism	4	2.71 × 10^−3^	
Carnitine shuttle	4	1.69 × 10^−2^	
Linoleate metabolism	3	1.79 × 10^−2^	
Vitamin E metabolism	5	1.65 × 10^−2^	Metabolism of vitamin E

^1^ Number of significant metabolites; ^2^ the adjusted *p*-Values were calculated to estimate the false discovery rate. Pathways with ≥3 significant metabolites and FDRs of <20% were considered.

## Data Availability

The NMR and LC–MS data that support the findings of this study will be available on the UCSD Metabolomics Workbench data repository with the identifier 3542.

## Supplementary Materials

The following supporting information can be download at: https://www.mdpi.com/article/10.3390/metabo12111081/s1. Figure S1. sPLS-DA, Figure S2. Heatmap of the mass spectrometry features selected by sPLS-DA, Figure S3. Multiblock integrative analysis for clinical, metabolomics and lipidomics data, Table S1. Clinical and biochemical characteristics of HC, ST and NASH patients, Supplementary sheets: the possible matches of selected LC-MS features [39,58,59].

## Abbreviations

AU-ROC, area under the receiver operating characteristic curve; BMI, body mass index; NMR, nuclear magnetic resonance spectroscopy; SD, standard deviation; CI, confidence interval; CIM, clustered image map; CV-AUC, cross-validated area under the ROC curve; FDR, false discovery rate; HC, health control; PBS, phosphate-buffered saline; PCA, principal component analysis; PC, principal component; IS, internal standard; PFAS, perfluorinated alkyl substances; SVD, singular value decomposition; ST, simple fatty liver or steatosis; UHPLC, ultra-high-performance liquid chromatography; HRMS, high-resolution mass spectrometry; VIP, variable importance in the projection; sPLS-DA, sparse partial least squares discriminant analysis.
