# Supplementary material for: Plasma Metabolomic and Lipidomic Profiling of Metabolic Dysfunction-Associated Fatty Liver Disease in Humans Using an Untargeted Multiplatform Approach
Source: Metabolites. 2022 Nov 8;12(11):1081. doi: 10.3390/metabo12111081 (PMC9693407; doi:10.3390/metabo12111081)
Supplement: Supplementary file 1 [file metabolites-12-01081-s001.zip › Supplemental Materials_MAFLD_11062022.pdf]

Supplemental Material and methods

Plasma metabolomics and lipidomics profiling of metabolic dysfunction-associated fatty liver disease in humans using an untargeted multiplatform approach

Xiangping Lin [1,2,*], Xinyu Liu [2], Mohamed N. Triba [1], Nadia Bouchemal [1], Zhicheng Liu [3], Douglas I. Walker [4], Tony Palama [1], Laurence Le Moyec [5], Marianne Ziol [6], Nada Helmy [7], Corinne Vons [7], Guowang Xu [2,†,*], Carina Prip-Buus [8,†] and Philippe Savarin [1,†,*]

Reagents and chemicals

HPLC grade solvent (acetonitrile, methanol and isopropanol) and formic acid were purchased from Merck KGaA (Darmstadt, Germany). Ammonium acetate and tert-butyl methyl ether (MTBE) was purchased from Merck KGaA (Darmstadt, Germany). Ultrapure water (H_2O) was collected from a Milli-Q system (Millipore, Billerica, MA). Internal standards (ISs) Carnitine C8:0-d3, Carnitine C16:0-d3, free fatty acid d3-FFA (C18:0), CA-d4, CDCA-d5, Phe-d5 and Trp-d5 were purchased from Cambridge Isotope (Tewksbury, MA), free fatty acid d3-FFA (C16:0) were purchased from C/D/N Isotopes Inc. (Pointe-Claire, Québec), triacylglycerol TAG(15:0/15:0/15:0) was purchased from Merck KGaA (Darmstadt, Germany), ceramide Cer(d18:1/17:0), lysophosphatidylcholine LPC(19:0), phosphatidylcholine PC(19:0/19:0), sphingomyelin SM(d18:1/12:0) were supplied by Avanti Polar Lipids (Alabaster, AL). ISs were prepared in methanol as stock solution with the follow concentrations and stored in -20°C before use: PC (19:0/19:0) 0.67 µg/mL, LPC (19:0) 0.33

μg/mL, SM (d18:1/12:0) 0.17 μg/mL, Cer(d18:1/17:0) 0.17 μg/mL, TAG (15:0/15:0/15:0) 0.53 μg/mL, d3-FFA (C16:0) 0.67 μg/mL, and d3-FFA(C18:0) 0.67 μg/mL.

Supplemental Samples Preparation, NMR and MS based analysis

For NMR profiling, randomized samples were thawed on ice and 250 μL plasma were prepared by adding 350 μL deuterated Phosphate-Buffered Saline (PBS) (containing NaN_3) to allow the deuterium frequency-field lock, after vortexed mix for 1 min, centrifuged for 10 min at 12 000× g at 4 °C, and finally 550 μL of the supernatant were transferred into a clean 5 mm NMR tube. A pooled QC sample was prepared with every 10 plasma samples as quality control, samples were prepared and analyzed as a single batch.

For LC-MS based metabolomic analysis, randomized plasma samples were thawed on ice and aliquots of each sample were pooled as QC samples. Then, 150 μL of sample were mixed with 600 μL ice-cold methanol containing ISs for deproteinization and metabolites extraction, after vortexed for 2 min, and centrifuged for 10 min at 16 000 × g, at 4 °C. Three aliquots with 50 μL supernatant each were lyophilized (prepared for positive mode analysis, negative mode analysis, and backup), then stock in -80°C. A pooled QC sample was prepared with every 10 plasma samples as quality control, samples were prepared and analyzed as a single batch.

For LC-MS based lipidomic analysis, randomized plasma samples were thawed on ice, and 20 μL aliquot of each sample were pooled as QC samples. For deproteinization, 40 μL plasma were mixed with 300 μL ice-cold methanol containing ISs, after vortexed 30s, 1 mL Methyl tert-butyl ether (MTBE) was added in the mixture and the samples were vibrated at room temperature for 1

h for lipids extraction. Then, 300 μL water were added followed by vortex 30 s and placed at 4°C for 10 min. After centrifugation at 14000 × g for 15 min at 4 °C, 2 aliquots of 400 μL supernatants were transferred into two new Eppendorf tubes, and the supernatants were lyophilized before storage at -80°C. A pooled QC sample was prepared for every 10 plasma samples as quality control, samples were prepared and analyzed as a single batch. Additional details are provided in the Supplemental Materials.

Before metabolomic analysis, the freeze-dried supernatant was re-dissolved with methanol/water (1:4, vol/vol) solvent. Five μL re-dissolved supernatant was injected for analysis on a Q Exactive HF (Thermo Fisher Scientific, Rockford, IL, USA) system coupled with an ACQUITY Ultra Performance Liquid Chromatography (UPLC, Waters Corporation, Manchester, U.K.). Column temperature and automatic sampler temperature were set at 60°C and 6°C, respectively. To maximize compounds detection, dual columns and dual polarity ionization mode were employed. For electrospray positive ion (ESI+) mode, ACQUITY UPLC BEH C8 column (130Å, 1.7 μm, 2.1 mm × 50 mm, Waters, Milford, MA, USA) was used to ensure the separation of weakly polar compounds such as carnitine and lipids. For electrospray negative ion (ESI-) mode, ACQUITY UPLC HSS T3 column (100Å, 1.8 μm, 2.1 mm × 50 mm, Waters, Milford, MA, USA) was used to ensure the retention and separation of polar compounds in reverse phase. The mobile phases include water containing 0.1% formic acid (A) and acetonitrile containing 0.1% formic acid (B) were used in electrospray positive ion (ESI+) mode. The flow rate was 0.40 mL/min and the total run time was 12 min. The elution program started with 5% B and was held for 0.5 min, then linearly increased to 40% B at 2 min and increased to 100% B at 8 min, maintained 2 min, then went back to 5% B in 0.1 min and kept for 1.9 min for post-equilibrium. For electrospray negative ion (ESI-)

mode, the mobile phases were water (A) and methanol/water (95:5, v/v) (B) containing 6.5 mM Ammonium bicarbonate. The flow rate was 0.40 mL/min and the total run time was 12 min. The elution program started with 2% B and was held for 0.5 min, then linearly increased to 40% B at 2 min and increased to 100% B in 8 min, maintained 2 min, then went back to 2% B in 0.1 min and kept for 1.9 min for post equilibrium. The resolutions of full scan MS and $ddMS_2$ were set at 120 000 and 60 000, respectively. The automatic gain control (AGC) target and maximum injection time in full scan MS settings were 1×10^6 and 200 ms, while their values were 1×10^5 and 50 ms in $ddMS_2$ settings. The TopN (N, the number of top most abundant ions for fragmentation) was set to 10, and collision energy was set to 15 eV, 30 eV and 45 eV. A heated ESI source was used for positive and negative ion mode. The spray voltage was set at 3.5 kV. The capillary temperature and aux gas heater temperature were set at 300 and 350 °C, respectively. Sheath gas and aux gas flow rate were set at 45 and 10 (in arbitrary units), respectively. The S-lens RF level was 50.

Before lipidomic analysis, the freeze-dried supernatant was re-dissolved in the mix of 20 μL solution A (chloroform : methanol, 2 : 1 (vol/vol)) and 80 μL solution B (water : isopropanol : acetonitrile, 5 : 30 : 65 (vol/vol/vol) containing 5mmol/L ammonium acetate), after vortexed for 1 min and centrifuged at 14 000 × g for 5 min at 4°C, lipidomic analysis was performed on the AB SCIEX TripleTOF™ 5600 plus mass spectrometer system (AB SCIEX™, Framingham, MA, USA) coupled with an ACQUITY Ultra Performance Liquid Chromatography (UPLC, Waters Corporation, Manchester, U.K.), equipped a reversed-phase UPLC ACQUITY C8 BEH column (130Å, 1.7 μm, 2.1 mm × 100 mm, Waters, Milford, USA), the column temperature was 55°C in electrospray positive and negative ionization (ESI+ and ESI-) modes. Acetonitrile : water, 6 : 4

(vol/vol) containing 10 mM ammonium acetate was used as mobile phase A. Isopropanol : acetonitrile, 9 : 1 (vol/vol) containing 10 mM ammonium acetate was used as mobile phase B. The flow rate was 0.26 mL/min, with the elution gradient as follows: 32% B was firstly maintained for 1.5 min, then linearly increased to 85% B in 14 min, linearly increased to 97% B from 15.5 min to 15.6 min, finally maintained for 2.4 min and followed by equilibration with 32% B in next 2 min. Data acquisition was performed both in full scan (with mass range from 200 to 1000m/z for ESI+, 90 to 1000m/z for ESI-) and IDA mode (with mass range from 100 to 1000m/z for ESI+, 50 to 1000m/z for ESI-). Mass spectrometry parameters were as follows: ion spray voltage, 5500V for ESI+ and -4500V for ESI-; curtain gas was 35 psi; declustering potential, full scan mode: 100V for ESI+ and -100V for ESI-, IDA mode: 80V for ESI+ and -100V for ESI-; collision energy, full scan mode: 10V for ESI+ and -10V for ESI-, IDA mode: 35V for ESI+ and -35V for ESI-, collision energy spread was 15 in ESI+ and ESI- mode; interface heater temperature, 500°C for ESI+ and 550°C for ESI-.

Supplemental Data analysis

NMR data

Processed 1D NOESY NMR data were used to build multivariate statistical models for classification of HC, ST and NASH patients. In our study, blood samples were collected with tube containing Sodium Citrate as anticoagulant during the preparation of blood plasma, consequently, NMR spectra region of Citrate (between 2.5 - 2.7ppm) were excluded for multivariate statistical analysis. Also, 1 HC and 8 NASH patients were excluded for multivariate statistical analysis due

to presence of strong sugar signal. The presence of this abnormal signal results to a very large variability on the spectra, and could impact the statistical analysis.

LC-MS data

The uniquely detected LC-MS peaks consisted of mass (m/z), retention time and ion response, referred as features here after. Clinical data, MS data statistical analysis and data visualization were performed using R (version 4.2.0 for macOS, https://www.r-project.org/) and RStudio Desktop (version 2022.02.3+492 for macOS, https://www.rstudio.com/). Before principal component analysis (PCA), clinical data were centered and scaled, MS data were normalized to the median of responses and pareto scaling, PCA was computed using the 'prcomp()' function in R with singular value decomposition (SVD) method. Visualization of the correlation matrix that highlights the most contributing variables for each component was accomplished using R package corrplot (https://github.com/taiyun/corrplot) on PCA loadings. PCA was computed using the 'prcomp()' function in R that use singular value decomposition (SVD), which examines the covariances / correlations between individuals, it has slightly better numerical accuracy compared with spectral decomposition, which examines the covariances / correlations between variables. sparse Partial Least Squares Discriminant Analysis (sPLS-DA) was performed using R package mixOmics [1].

R function 'lm()' was used to fit linear regression models to test the relationship between plasma TGs and liver TGs, and also used to test the relationship between selected metabolomics or lipidomics features and liver TGs. MS features were annotated by a computational match to metabolite or lipid compounds using R package xmsAnnotator, briefly, it is a Network-Based

computational compounds annotation pipeline, which integrates a multistage clustering algorithm that uses detected mass (*m/z*), retention time (rt), feature response (peak intensity), mass defect, adduct patterns, and metabolic pathway associations, to match *m/z* features to compounds databases [2]. The mass search tolerance for databases matching was set at ± 5 ppm mass tolerance for metabolomics data, and at ± 10 ppm for lipidomics data (Supplementary sheets).

Supplemental results

Table S1. Clinical and biochemical characteristics of HC, ST and NASH patients.

Characteristics	HC (n=19)	NAFL (n=39)	NASH(n=24)	*p*-value
Age (year)	32.2 (8.7)	36.2 (10.2)	41.7 (10)†	< 0.01
Gender (♂/♀)	(1/18)	(5/34)	(10/14)	-
BMI (kg/m^2)	42.9 (4.6)	45.3 (5.1)	44.5 (5.3)	NS
ALT (IU/L)	18.7 (7.3)	28.5 (20.3)‡	50.0 (28.3)†	< 0.001
AST (IU/L)	20.1 (5.1)	25.3 (11.7)‡	34.7 (15.5)†	< 0.002
GGT (IU/L)	19.9 (5.5)	35.2 (38.5)‡	47.2 (31.9)†	< 0.001
ALP (IU/L)	68.9 (16.3)	74.1 (18.9)	69.0 (24.4)	NS
TG (mmol/L)	1.0 (0.5)	1.3 (0.6)‡	2.3 (1.5)†	< 0.001
Cholesterol (mmol/L)	4.8 (0.9)	4.7 (1.0)	5.0 (1.1)	NS
Fasting blood Glucose (mmol/L)	5.0 (0.6)	5.3 (1.2)‡	6.7 (2.6)†	< 0.002
Fasting Insulin (pmol/L)	71.5 (39.3)	142.2 (109.0)*	171.8 (150.6)†	< 0.02
HOMA-IR (a.u.)	2.4 (1.3)	5.2 (4.5)*	7.2 (5.6)†	< 0.004
Triglycerides (mg/g of liver)	6.1 (2.9)	16.0 (12.4)*‡	25.5 (14.6)†	< 0.001
Phospholipids (mg/g of liver)	0.8 (0.7)	0.8 (0.6)	0.8 (0.4)	NS
Diacylglycerols (mg/g of liver)	0.6 (0.4)	0.7 (0.3)	0.9 (0.5)†	< 0.04

Data are expressed as “mean (SD)”. *p*-value were calculated with Kruskal-Wallis test, Dunn’s Multiple Comparison Test have been carried out when the Kruskal-Wallis test was significant. This will compare the pairs of groups, but is statistically more sensible than doing pairwise Mann-Whitney tests, significant differences between groups ($p < 0.05$) are represented by *: HC - ST, †: HC - NASH, and ‡: ST - NASH. BMI: Body Mass Index, TG: Triglycerides, AST: Aspartate transaminase, ALT: Alanine transaminase, GGT: Gamma-Glutamyl Transferase, ALP: Alkaline phosphatase, HOMA-IR: Homeostatic Model Assessment for Insulin Resistance, is a method for assessing β-cell function and Insulin Resistance (IR), NS: Non-Significant, HC: Normal Liver, ST: Nonalcoholic fatty liver or steatosis, NASH: Non-Alcoholic SteatoHepatitis, SD: Standard deviation, a.u.: arbitrary unit.

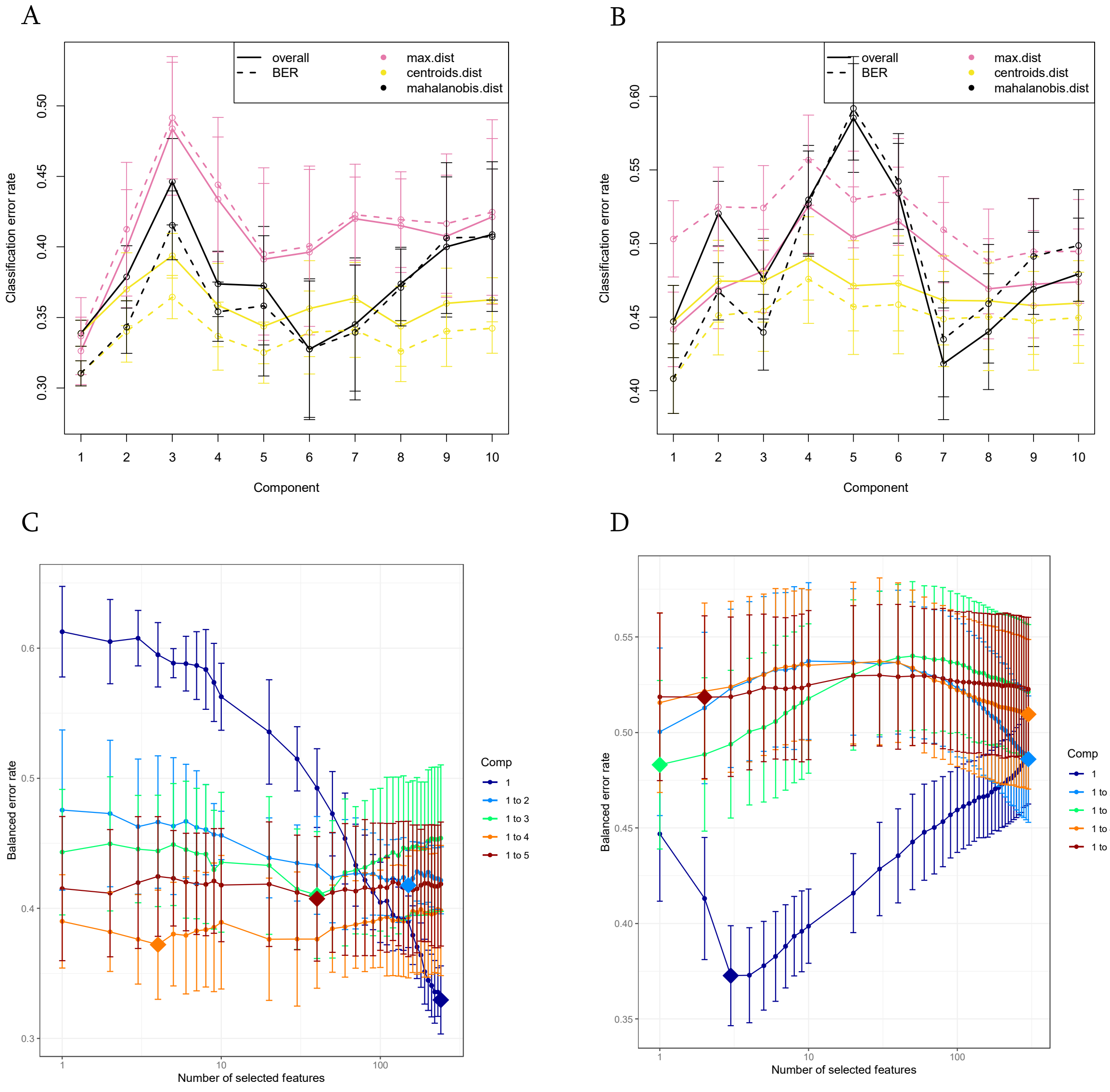

Figure S1. sPLS-DA. (A) outcome of performance evaluation across all ten components on the metabolomics data, (B) outcome of performance evaluation across all ten components on the lipidomics data, (C) output of variable number tuning on the metabolomics data, (D) output of variable number tuning on the lipidomics data. All evaluation above were performed based on 200 times repeated 10-fold cross validation.

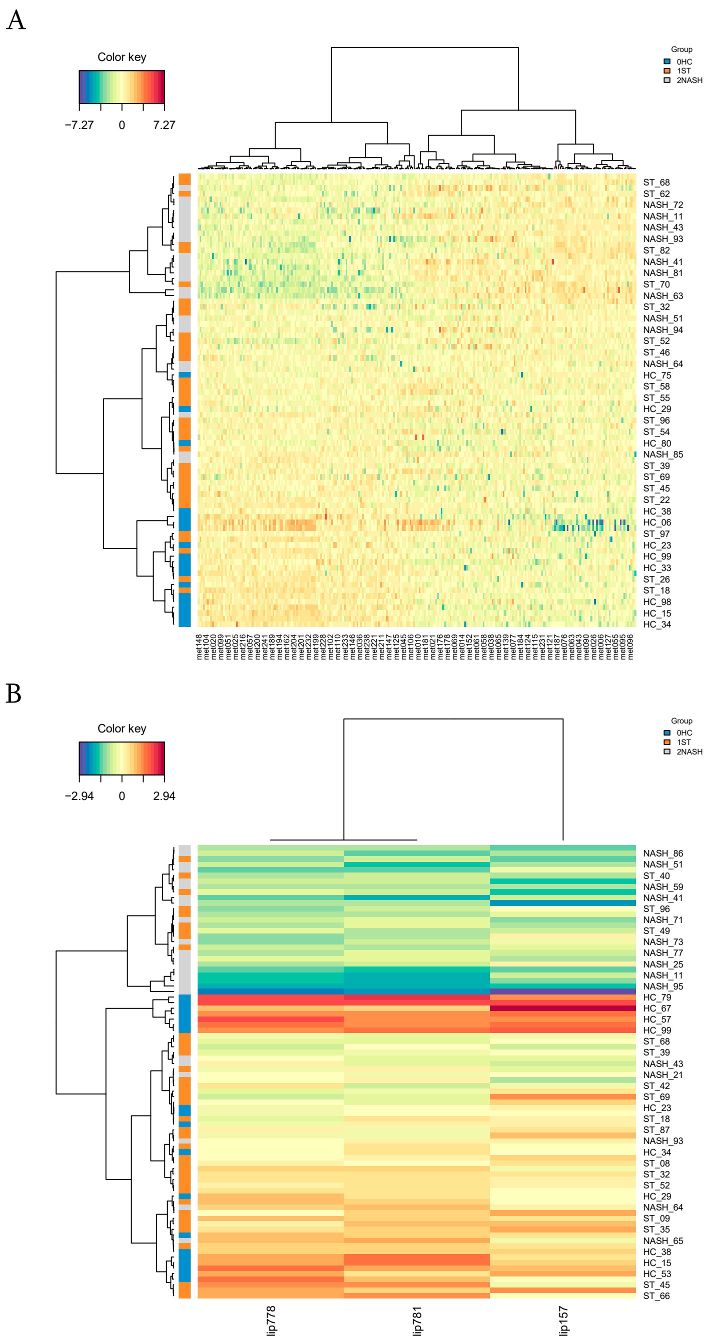

Figure S2. Heatmap of the mass spectrometry features selected by sPLS-DA. (A) heatmap on metabolomics data, (B) heatmap on lipidomics data. A hierarchical clustering based on the responses of the selected features, with samples in rows colored according to the stage of MAFLD, Euclidean distance with Complete agglomeration method was employed. Features name were coded with "metxxx" for metabolomics and "lipxxx" for lipidomics, the possible matches in database were provided in the Supplementary sheets.

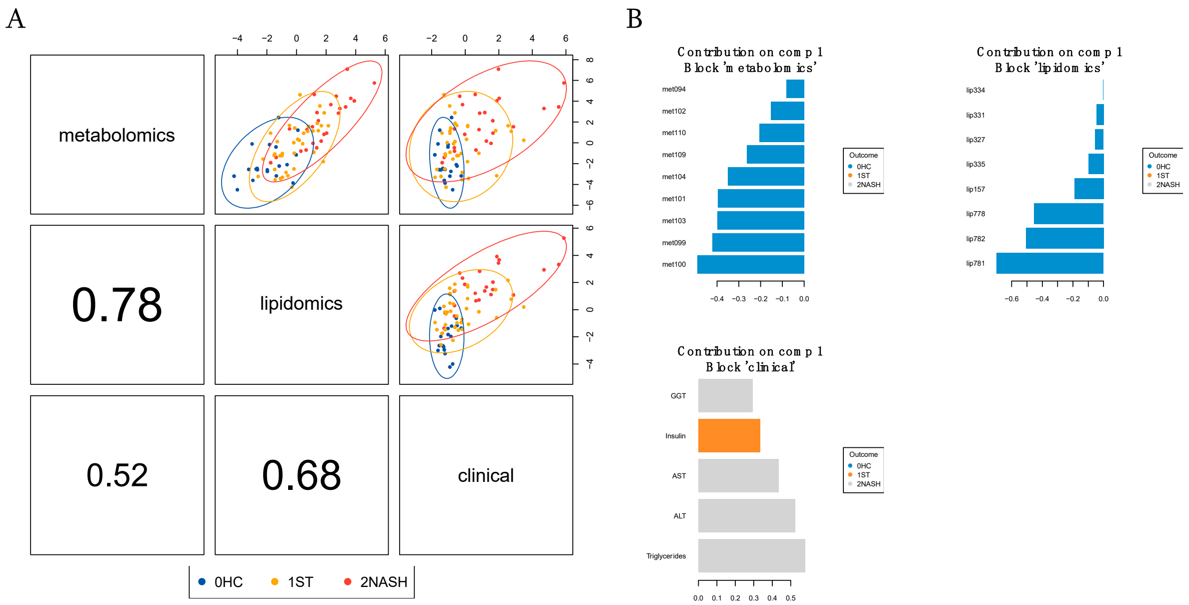

Figure S3. Multiblock integrative analysis for clinical, metabolomics and lipidomics data. (A) Diagnostic plot from multiblock sPLS-DA. Samples are represented based on the first component for each data set (clinical, metabolomics and lipidomics). Samples are colored by stages of MAFLD and 95% confidence ellipse plots are represented. (B) Loading plot for the variables selected by multiblock sPLS-DA performed on the first component. The most important variables are ordered from bottom to top based on the absolute value of their coefficients. Colors indicate the stages of MAFLD for which the median expression value is the highest for each feature, selected features name were coded with "metxxx" for metabolomics and "lipxxx" for lipidomics, the possible matches were provided in the Supplementary sheets.

References

[1] Rohart F, Gautier B, Singh A, Lê Cao K-A. mixOmics: An R package for 'omics feature selection and multiple data integration. PLoS Comput Biol 2017;13:e1005752. https://doi.org/10.1371/journal.pcbi.1005752.

[2] Uppal K, Walker DI, Jones DP. xMSannotator: An R Package for Network-Based Annotation of High-Resolution Metabolomics Data. Anal Chem 2017;89:1063–7. https://doi.org/10.1021/acs.analchem.6b01214.
